# Supplementary material for: GABA uptake transporters support dopamine release in dorsal striatum with maladaptive downregulation in a parkinsonism model
Source: Nat Commun. 2020 Oct 2;11:4958. doi: 10.1038/s41467-020-18247-5 (PMC7532441; doi:10.1038/s41467-020-18247-5)
Supplement: Supplementary file 3 — Reporting Summary [file 41467_2020_18247_MOESM3_ESM.pdf]

nature research

Corresponding author(s): Dr Bradley Roberts
Prof Stephanie Cragg

Last updated by author(s): Aug 4, 2020

Reporting Summary

Nature Research wishes to improve the reproducibility of the work that we publish. This form provides structure for consistency and transparency in reporting. For further information on Nature Research policies, see our Editorial Policies and the Editorial Policy Checklist.

Statistics

For all statistical analyses, confirm that the following items are present in the figure legend, table legend, main text, or Methods section.

n/a	Confirmed	
☐	☒	The exact sample size (*n*) for each experimental group/condition, given as a discrete number and unit of measurement
☐	☒	A statement on whether measurements were taken from distinct samples or whether the same sample was measured repeatedly
☐	☒	The statistical test(s) used AND whether they are one- or two-sided<br>*Only common tests should be described solely by name; describe more complex techniques in the Methods section.*
☐	☒	A description of all covariates tested
☐	☒	A description of any assumptions or corrections, such as tests of normality and adjustment for multiple comparisons
☐	☒	A full description of the statistical parameters including central tendency (e.g. means) or other basic estimates (e.g. regression coefficient) AND variation (e.g. standard deviation) or associated estimates of uncertainty (e.g. confidence intervals)
☐	☒	For null hypothesis testing, the test statistic (e.g. *F*, *t*, *r*) with confidence intervals, effect sizes, degrees of freedom and *P* value noted<br>*Give P values as exact values whenever suitable.*
☒	☐	For Bayesian analysis, information on the choice of priors and Markov chain Monte Carlo settings
☒	☐	For hierarchical and complex designs, identification of the appropriate level for tests and full reporting of outcomes
☒	☐	Estimates of effect sizes (e.g. Cohen's *d*, Pearson's *r*), indicating how they were calculated

Our web collection on statistics for biologists contains articles on many of the points above.

Software and code

Policy information about availability of computer code

Data collection	Voltammetry data were collected using using Axoscope 11.0 (Molecular Devices). Electrophysiology data were acquired using Clampfit 10.4.1.4 software (Molecular Devices). Confocal microscopy images were acquired using Zen Black 2.3 software (Zeiss). Western Blot experiments were acquired using Image Lab 5.1 software (BioRad). Details on data collection for each experiment are provided in the Methods.
Data analysis	Voltammetry data were analyzed using Axoscope 11.0 (Molecular Devices) and Microsoft Excel (2013). Electrophysiology data were analyzed using Clampfit 10.4.1.4 software (Molecular Devices). Confocal microscopy images were analyzed in Image J 1.5 (NIH) and ZEN (blue edition v.2.3; Zeiss). Western Blot experiments were analyzed using Image Lab 5.1 software (BioRad).

For manuscripts utilizing custom algorithms or software that are central to the research but not yet described in published literature, software must be made available to editors and reviewers. We strongly encourage code deposition in a community repository (e.g. GitHub). See the Nature Research guidelines for submitting code & software for further information.

Data

Policy information about availability of data

All manuscripts must include a data availability statement. This statement should provide the following information, where applicable:

- Accession codes, unique identifiers, or web links for publicly available datasets
- A list of figures that have associated raw data
- A description of any restrictions on data availability

Source data underlying all figures has been provided in the Source Data file.

Field-specific reporting

Please select the one below that is the best fit for your research. If you are not sure, read the appropriate sections before making your selection.

☒ Life sciences ☐ Behavioural & social sciences ☐ Ecological, evolutionary & environmental sciences

For a reference copy of the document with all sections, see nature.com/documents/nr-reporting-summary-flat.pdf

Life sciences study design

All studies must disclose on these points even when the disclosure is negative.

Sample size	Formal sample size calculations were not performed. Our previous experiments with similar techniques (Janezic et al. PNAS 2013; Threlfell et al., Neuron 2012) suggest that sample sizes of 3–7 animals (biological replicates) for each experimental condition are sufficient to detect drug-induced and genotype changes and this information is balanced against the ethical requirement to use as few animals as possible.
Data exclusions	No data were excluded from this study.
Replication	No replication attempts were made.
Randomization	For all experiments, randomization was not applicable as the groups were determined by genotype.
Blinding	Blinding was not feasible in this study, due to the need for the experimenter to apply buffers and drugs to the slice during each experiment and to subsequently match timings and calibration factors to the data during analysis.

Reporting for specific materials, systems and methods

We require information from authors about some types of materials, experimental systems and methods used in many studies. Here, indicate whether each material, system or method listed is relevant to your study. If you are not sure if a list item applies to your research, read the appropriate section before selecting a response.

Materials & experimental systems

n/a	Involved in the study	
☐	☒	Antibodies
☒	☐	Eukaryotic cell lines
☒	☐	Palaeontology and archaeology
☐	☒	Animals and other organisms
☒	☐	Human research participants
☒	☐	Clinical data
☒	☐	Dual use research of concern

Methods

n/a	Involved in the study	
☒	☐	ChIP-seq
☒	☐	Flow cytometry
☒	☐	MRI-based neuroimaging

Antibodies

Antibodies used	Rabbit anti-TH (1:2000, Sigma-Aldrich, ab112)<br>Rabbit anti-GAT1 (1:1,000, Synaptic Systems, 274102)<br>Rabbit anti-GAT3 (1:250, Millipore/Chemicon, AB1574)<br>Rabbi anti-NeuN (1:500, Biosensis, R-3770-100)<br>Guinea pig anti-S100β (1:2,000, Synaptic Systems, 287004)<br>Rat anti-GFP that also recognizes eYFP (1:1,000, Nacalai Tesque, 04404-84)<br>Guinea-pig anti-parvalbumin (1:1,000, Synaptic Systems, 195004)
Validation	Commercial antibodies were validated by the manufacturer.

Animals and other organisms

Policy information about studies involving animals; ARRIVE guidelines recommended for reporting animal research

Laboratory animals	C57Bl6/J mice (male, 6-8 weeks, Charles River), DAT-IRES-Cre mice and PV-Cre mice on a C57Bl6/J background (male, 4-5 weeks), and alpha-synuclein overexpressing (SNCA-OVX) mice (male and female, 11-12 weeks) were used in this study.
Wild animals	No wild animals were used in this study.
Field-collected samples	No field collected samples were used in the study.

Ethics oversight	All procedures were performed in accordance with the Animals in Scientific Procedures Act 1986 (Amended 2012) with ethical approval from the University of Oxford, and under authority of a Project Licence granted by the UK Home Office.

Note that full information on the approval of the study protocol must also be provided in the manuscript.
